# Effect of premixed and sequential intrathecal fentanyl on spinal block characteristics

**DOI:** 10.6026/973206300221769

**Published:** 2026-03-31

**Authors:** Priyanka Gupta, Utsav Sharma, Deepak Gupta, Lokesh Bansal, Payal Jain

**Affiliations:** 1 Department of Anesthesiology, S.R.V.S Medical College, Shivpuri, Madhya Pradesh, India; 2 Department of Anaesthesiology, DR Laxminarayan Pandey Government Medical College, Ratlam, India; 3 Department of Anaesthesiology, Indraprastha Apollo Hospital, New Delhi, India; 4 Department of Anaesthesiology, ESIC Medical College & Hospital, Faridabad

**Keywords:** Spinal anaesthesia, subarachnoid block, hyperbaric bupivacaine, Intrathecal fentanyl, premixed technique, Sequential technique

## Abstract

The optimal method of intrathecal fentanyl administration with hyperbaric bupivacaine remains uncertain, as premixed and sequential techniques may influence block characteristics differently. In this prospective observational comparative study, 42 ASA I-II adults (21 per group) received fentanyl 0.5 ml with hyperbaric bupivacaine 2.5 ml either premixed or sequentially. Sequential administration produced faster sensory onset at T10 (5.00±1.37 vs 6.48±1.32 min) and motor onset (6.10±1.37 vs 8.00±1.30 min) with longer sensory and motor regression times. Time to first rescue analgesia was also longer (205.33±12.08 vs 175.38±14.41 min), suggesting earlier onset and prolonged block with the sequential technique.

## Background:

Spinal anaesthesia is still a very common choice for infra-umbilical and lower limb surgery because onset is fast, airway handling is avoided and block reliability is usually good [1]. Hyperbaric bupivacaine remains the routine drug for SAB because gravity-dependent spread makes the block more predictable in many setups [2]. To optimise the block and extend postoperative analgesia, intrathecal opioids are commonly added to local anaesthetic solutions [3]. Evidence shows intrathecal opioid with local anaesthetic combinations can improve postoperative pain outcomes, but side effects can increase depending on opioid and dose [4]. Fentanyl is widely used because it is lipophilic and gives rapid spinal analgesia with relatively limited late respiratory depression when compared to hydrophilic opioids [5]. The practical controversy is not "whether fentanyl works" but how it should be given with heavy bupivacaine, premixed in one syringe or sequential in two syringes [6]. Mixing fentanyl with hyperbaric bupivacaine can reduce the final injectate density because fentanyl solution is relatively hypobaric and even small density shifts (around the 0.0006 g/mL range) may theoretically alter intrathecal spread patterns [1].

Clinical trials have shown non-identical block behaviour between premixed and sequential techniques, but findings vary by population and method [7]. In an RCT in lower limb surgeries, succedent/separate administration (heavy bupivacaine then fentanyl) showed faster onset and longer duration compared with premixed in the published comparison [8]. More recent randomised work with multiple groups again suggests the sequence "bupivacaine then fentanyl" may perform best for onset, duration and rescue analgesia timing [9]. Therefore, it is of interest to evaluate and report the effect of premixed versus sequential intrathecal fentanyl with hyperbaric bupivacaine on block characteristics and postoperative analgesia, to provide additional clinical evidence for technique selection.

## Material and Methods:

This was a prospective observational comparative study conducted in admitted patients posted for infra-umbilical and lower limb surgeries under spinal anaesthesia (subarachnoid block). After Ethics Committee approval, eligible patients were explained the procedure and written informed consent was taken. Adults aged >20 years, ASA I-II, height >150 cm and BMI 20-35 kg/m^2^ were included. Patients refusing consent, having uncontrolled arterial hypertension or coronary heart disease, contraindication to SAB (raised intracranial pressure, coagulation disorder, lumbar skin infection, hypovolaemia, marked spinal deformity), allergy to local anaesthetics, sinus bradycardia (HR <60 bpm) or 2nd/3rd degree heart block were excluded. Sample size was 42, with 21 patients per group, calculated to detect a clinically significant 1-minute difference in onset of block at alpha 0.05 and power 90%. Patients were observed and categorised into two groups based on the technique used in routine practice: Group A (Premixed) received 0.5% hyperbaric bupivacaine 2.5 ml + fentanyl 0.5 ml mixed in a single syringe and injected intrathecally, while Group B (Sequential) received fentanyl 0.5 ml and 0.5% hyperbaric bupivacaine 2.5 ml in separate syringes given sequentially intrathecally. All patients were kept fasting for at least 6 hours. In the OT, standard monitors (ECG, pulse oximetry, non-invasive BP) were applied and baseline vitals recorded. IV access was secured and crystalloid co-loading with Ringer lactate 6 ml/kg was given. Under strict asepsis with patient sitting, lumbar puncture was performed at L3-L4 or L4-L5 after local infiltration with 2% lignocaine and study drugs were administered as per group technique, then patient made supine. Sensory block was assessed by sterile pin-prick every 2 minutes for the first 10 minutes, then every 10 minutes till maximum level achieved; onset of sensory block was defined as loss of sensation at T10 dermatome, highest sensory level and sensory regression time were recorded. Motor block onset and motor regression time were recorded using the standard motor assessment used in the department (same method for both groups). Time to first requirement of rescue analgesia (minutes) was noted from establishment of SAB. Data were analysed using SPSS version 16.0. Continuous variables were expressed as mean ± SD and compared using unpaired t-test, categorical variables as number (%) and analysed using Chi-square or Fisher exact test as appropriate, with p<0.05 taken as statistically significant.

## Results and Discussion:

Baseline characteristics were comparable between the two groups (Table 1 - see PDF). The mean age was 48.19±15.34 years in the premixed group and 42.95±17.06 years in the sequential group with no significant difference (p>0.05), male proportion was 81.0% vs 71.4% and ASA II distribution was 61.9% vs 66.7%, again not significant. Sensory block onset at T10 was slower with premixed administration (6.48±1.32 min) compared to sequential (5.00±1.37 min) and this difference was significant (p=0.001) (Table 2 - see PDF). Highest sensory level at T6 was achieved in 76.2% of premixed and 85.7% of sequential cases with borderline significance (p=0.05, Table 2 - see PDF). Sensory regression time was shorter in the premixed group (88.19±4.60 min) than sequential (91.05±3.80 min) with significance (p=0.03, Table 2 - see PDF). Motor block onset was also slower in premixed (8.00±1.30 min) than sequential (6.10±1.37 min) significant (p=0.001) and motor regression time was shorter in premixed (131.10±9.41 min) compared to sequential (138.81±11.03 min), also significant (p=0.01) (Table 3 - see PDF). Time to first requirement of rescue analgesia was significantly lower with premixed administration (175.38±14.41 min) compared to sequential administration (205.33±12.08 min) with p=0.0001 (Table 4, Figure 1 - see PDF), suggesting earlier analgesic demand in the premixed technique. Time endpoints and assessment intervals were prespecified and applied uniformly, consistent with outcome-reporting guidance that emphasises clear definition, timing, and analysis of trial outcomes to improve transparency and interpretability [10]. Baseline demographic profile (age, sex distribution, ASA grade) was comparable across the two techniques in our cohort, so the observed differences in block onset and duration are more likely technique-related rather than patient mix. Published work using hyperbaric bupivacaine with intrathecal opioid adjuvants reports comparable onset and regression patterns, supporting that our timing endpoints are within expected clinical ranges despite differences in definitions and assessment intervals [11].

In our study, sequential administration produced a faster onset of sensory block at T10 (5.00 ± 1.37 min) than premixed injection (6.48 ± 1.32 min). Previous comparative and randomized studies have also shown that intrathecal technique and drug distribution characteristics can influence early block onset and spread, supporting the concept that method of administration may alter initial block dynamics [12]. Similar findings were reported in elective caesarean section, where sequential dosing showed earlier onset than premixed: sensory onset median 4 min (IQR 3-4) vs 4 min (IQR 4-5) and motor onset median 5 min (IQR 4-5) vs 5 min (IQR 4.5-6). First rescue analgesia was later with sequential (287.909 ± 15.255 min) than premixed (261.39 ± 25.378 min) [13]. An RCT reported that sequential (two-syringe) administration achieved a higher cephalad spread (higher peak sensory level) than the premixed technique (T6 in sequential groups vs T8 in premixed), indicating that technique can influence maximal block height, though the magnitude may vary across protocols [6]. Analgesic duration in our study was significantly longer with sequential technique (205.33±12.08 vs 175.38±14.41 min), meaning later analgesic requirement with the two-syringe method. In a double-blinded-RCT, separate injection of hyperbaric bupivacaine with fentanyl and morphine produced slightly better postoperative analgesia with lower VAS pain scores than the premixed technique, while block height and regression times were similar between groups [14]. Experimental and clinical LSCS evidence also suggests that premixed versus sequential intrathecal opioid with hyperbaric bupivacaine can show broadly similar block profiles, with only small shifts in timing endpoints, supporting that technique effects may be present but not always large across all measures [15]. In a controlled trial in caesarean section, sequential intrathecal hyperbaric bupivacaine plus fentanyl achieved the target sensory level faster and produced a longer sensory block than premixed administration, with similar hypotension and comparable neonatal outcomes [16]. Intrathecal fentanyl is known to prolong analgesia and improve block quality, but opioid-related side effects remain relevant. A randomized clinical trial reported a significantly higher incidence of pruritus in patients receiving intrathecal fentanyl with hyperbaric bupivacaine, while maternal and neonatal outcomes remained comparable [17]. Contemporary evidence-based guidance indicates that neuraxial opioid selection influences the balance between onset characteristics, duration of postoperative analgesia, and side effects, with longer-acting agents such as intrathecal morphine providing prolonged analgesia when combined with lipophilic opioids like fentanyl [18]. Our findings show that sequential administration (two syringes) produced earlier sensory and motor onset with longer sensory or motor regression times and later first rescue analgesia compared with premixing in one syringe. The direction of change matches multiple comparative studies, while absolute timings can vary depending on dose, population and how onset/regression endpoints are defined.

## Conclusion:

Premixed administration showed slower onset and earlier regression of sensory and motor block. Sequential administration delayed the first requirement of rescue analgesia and gave a more prolonged effective block. Sequential technique may be preferred when longer duration and earlier onset are desired while premixed remains a simpler but shorter acting option.
